# Lumbar radiculopathy caused by foraminal stenosis in rheumatoid arthritis

**DOI:** 10.3109/03009734.2010.526722

**Published:** 2011-04-12

**Authors:** Tomoaki Koakutsu, Naoki Morozumi, Yutaka Koizumi, Yushin Ishii

**Affiliations:** Department of Orthopaedic Surgery, Nishitaga National Hospital, Sendai, Japan

**Keywords:** Diagnosis, foraminal stenosis, lumbar spine, radiculopathy, rheumatoid arthritis, surgery

## Abstract

**Study design:**

Case-series study.

**Objective:**

To describe the clinical presentation, characteristic findings of imaging studies, and treatment of lumbar radiculopathy caused by foraminal stenosis in rheumatoid arthritis.

**Background:**

Lumbar lesions in rheumatoid arthritis are relatively rare, with a limited number of systemic reports.

**Methods:**

Six patients with lumbar radiculopathy caused by foraminal stenosis in rheumatoid arthritis were treated. The patients were all women with a mean age of 69 years and mean rheumatoid arthritis duration of 15 years. The medical records and imaging studies of all patients were reviewed.

**Results:**

The affected nerve roots were L4 in four patients and L3 in two patients. Foraminal stenosis was not demonstrated in magnetic resonance images in four of the six patients. Selective radiculography with nerve root block reproduced pain, manifested blocking effect, and demonstrated compression of the nerve root by the superior articular process of the lower vertebra in all patients. Conservative treatment was performed on one patient, and surgery was conducted for the rest of the five patients; radiculopathy was improved in all patients.

**Conclusions:**

Lumbar foraminal stenosis is a characteristic pathology of rheumatoid arthritis, and should be kept in mind in the diagnosis of lumbar radiculopathy. Selective radiculography is useful in the diagnosis of affected nerve roots.

## Introduction

Although cervical lesions are well known as spinal involvement of rheumatoid arthritis (RA), lumbar lesions are relatively rare, with a limited number of systemic reports (1–3). Most of the cases which have been reported so far are spinal canal stenosis complicated with neurological symptoms caused by vertebral collapse secondary to vertebral body lesions (4,5) or by segmental instability subsequent to facet joint lesions (6,7); thus very few reports have been published about foraminal stenosis (8). We retrospectively reviewed six cases of lumbar radiculopathy in RA that resulted from foraminal stenosis to describe the clinical presentation, characteristic findings of imaging studies, and treatment of this condition. Particularly, we compared the usefulness in the diagnosis of foraminal stenosis between magnetic resonance imaging (MRI) and selective radiculography (SRG) with nerve root block (9).

## Patients and methods

The subjects were six patients with RA who met the diagnostic criteria of the American Rheumatism Association and who were treated at our hospital in 2003–2005 for lumbar radiculopathy caused by foraminal stenosis. The patients were all females, aged 57 to 75 years (mean 69 years) with 2 to 28 years of duration of RA (mean 15 years). According to Steinbrocker's classification (10), RA staging was evaluated as Stage 2 in one patient, Stage 3 in one patient, and Stage 4 in four patients. Their functional classification was assessed as Class 3 in four patients and Class 4 in two patients. Mutilating deformities of the limbs were observed in four patients. All hospital and out-patient records as well as the related radiographs (plain X-rays, myelograms, postmyelographic CT scans, MRI, and SRG) were reviewed to study clinical presentation, characteristic findings of imaging studies, and treatment.

Lumbar radiculopathy in RA was diagnosed on the basis of neurological examination findings and imaging of the affected nerve roots. Severity was rated according to the criteria for evaluation of low back pain proposed by the Japanese Orthopaedic Association (JOA score). The therapeutic modality (with or without surgery; surgical procedures) was noted, and recovery rate was determined from pre- and postoperative JOA scores by Hirabayashi's method (11).

Generally, it is difficult to diagnose RA-specific lumbar lesions from imaging alone, since lesions of spondylosis deformans and/or lesions of osteoporosis associated with RA are also found. However, we defined RA-specific lesions as follows: rheumatoid nodules in the vertebral bodies (4); vertebral collapse without previous trauma (5); disc space narrowing without osteophyte formation; end-plate erosion (2); and segmental instability associated with facet joint destruction (6,7). Thus the presence of these lesions had to be confirmed. Since coexisting cervical myelopathy may affect neurological diagnosis, the presence of RA cervical lesions (atlantoaxial subluxation, vertical subluxation, and subaxial subluxation) was examined in plain X-rays of the cervical spine. Lumbar spinal canal stenosis was assessed by MRI or myelography/postmyelographic CT scans. Foraminal stenosis was diagnosed on MRI (sagittal and transverse sections through the intervertebral foramen) and SRG, and their diagnostic usefulness was compared.

## Results

All of the patients complained of pain over the buttock to the anterior surface of the thigh. Pain was worsened in the sitting and standing positions but was mitigated in the recumbent position. The L2, L3, and L4 dermatomes were suspected to be the responsible nerve roots. However, complications such as cervical myelopathy and articular lesions of RA in the lower limbs made it difficult to identify the responsible nerve roots on the basis of neurological findings alone. From combined neurological and imaging findings, responsible nerve roots were finally identified as L3 and L4 in two and four patients, respectively. Radiculopathy was unilateral in five patients and bilateral in one patient. Cauda equine syndrome associated with radiculopathy was diagnosed in only one patient.

Conservative treatment alleviated the symptoms of one patient; five patients were treated by surgical intervention. Preoperative JOA score was 8.6 ± 6.3 (range 5–13). Three patients were treated by posterior decompression for the affected nerve root. A patient with severe low back pain and another patient with segmental instability in multi-levels were treated by posterior decompression and posterolateral fusion with instrumentation. Although radiculopathy was improved in all patients, one patient was re-operated for radiculopathy that had developed in the contralateral side after the first operation (Table I). Their clinical courses were monitored postoperatively for 26.0 ± 3.5 months (range 12–48). Postoperative JOA score was improved to 15.2 ± 7.5 (range 8–20) with a recovery rate of 32.2% ± 3.7% (range 12.5%–52.6%). Bone fusion was achieved in all patients who were treated by posterior decompression and posterolateral fusion with instrumentation.

**Table I.** Patients' data.

Case	Gender, age (years)	Duration of RA (years)	Mutilating	Classification	Affected nerve root	Treatment
1	F, 74	17	+	Stage IV, Class 4	Left L4 → Right L4 ^a^	Decompression
2	F, 69	28	+	Stage IV, Class 4	Right L4	Decompression
3	F, 57	25	+	Stage III, Class 3	Right L3	Decompression and fusion
4	F, 74	2	+	Stage IV, Class 3	Right L4	Conservative
5	F, 65	26	-	Stage IV, Class 3	Left L3	Decompression and fusion
6	F, 75	3	-	Stage II, Class 3	Bilateral L4	Decompression

^a^ Re-operated.

**Figure 1.**
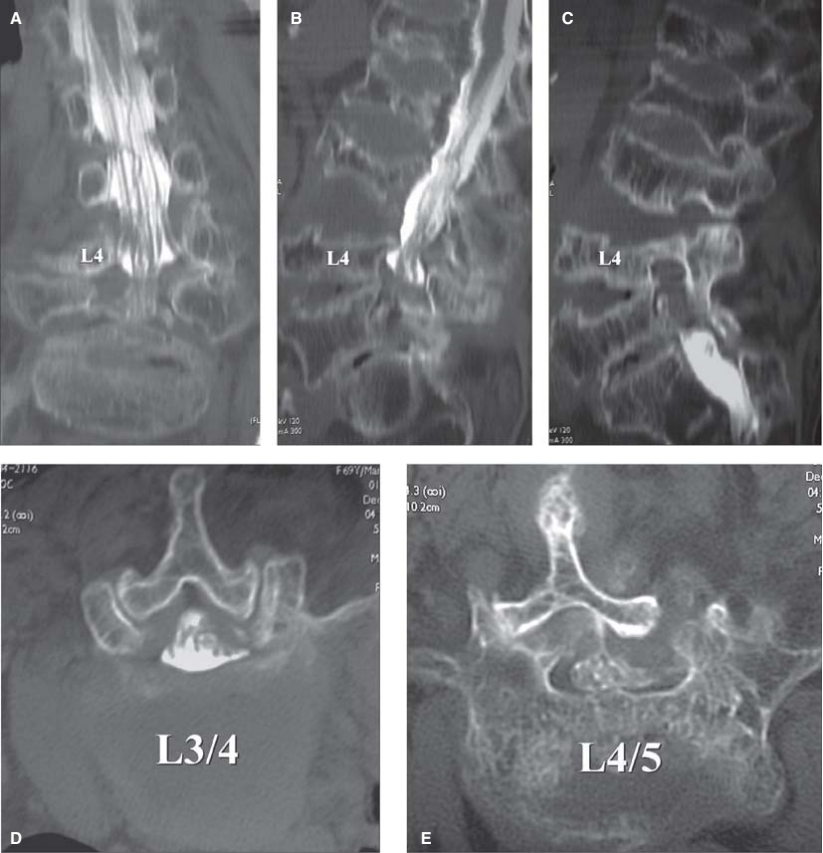
A 69-year-old women, with right L4 radiculopathy as revealed by postmyelographic CT scan. A: In the coronal section, bilateral L4 roots were well depicted. B: In the sagittal section, osteoporosis is evident; multiple vertebral collapse and end-plate irregularity are observed despite no history of trauma. C: In the sagittal section through the right foramen, foraminal stenosis is not obvious. D: In the transverse section (L3/4), spinal canal stenosis is very mild. E: In the transverse section (L4/5), prominent L4/5 facet joint destruction and lateral dislocation are noted.

**Figure 2.**
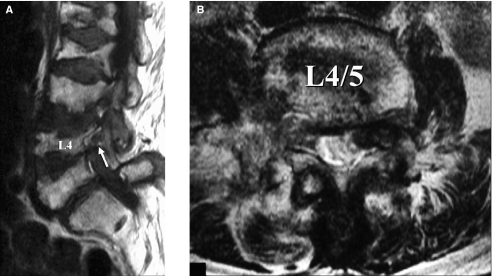
Magnetic resonance imaging (MRI). A: In the sagittal section (right L4/5 foramen; T1-weighted image), foraminal stenosis is not obvious (arrow). B: In the transverse section (L4/5 foramen; T2-weighted image) as well, foraminal stenosis is not evident.

**Figure 3.**
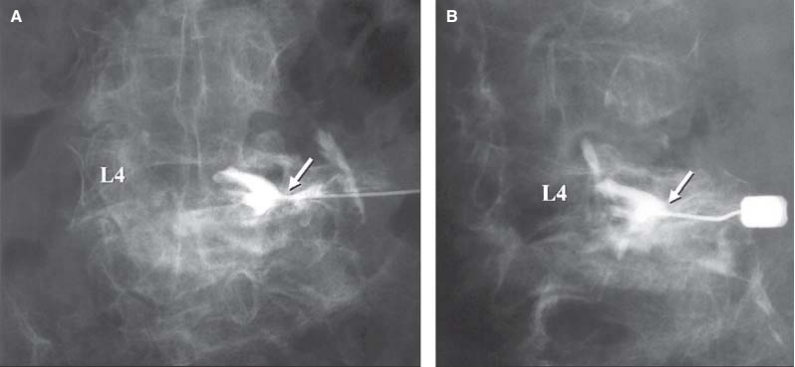
Selective radiculography (SRG) (right L4 nerve root). A: Posteroanterior view. B: Oblique view. The right L4 nerve root is compressed from underneath by the L5 superior articular process (arrow). Pain reproduction at the time of nerve puncture and nerve block induction were confirmed.

Plain X-rays of the cervical spine revealed RA lesions (atlantoaxial subluxation, vertical subluxation, and subaxial subluxation) in four of the six patients. Plain X-rays of the lumbar spine, CT, and MRI found the following RA lesions: vertebral collapse without previous trauma in three patients; disc space narrowing without osteophyte formation in three; end-plate erosion in six; and segmental instability associated with facet joint destruction in four (anterior spondylolisthesis in two; lateral spondylolisthesis in two; includes overlapping lesion). In all patients, severe osteoporosis was evident, although MRI revealed no obvious rheumatoid nodules in the vertebral bodies. Excluding the one patient with cauda equine syndrome, spinal canal stenosis was mild in the MR images and/or the myelograms. This finding led us to suspect the presence of foraminal stenosis. Foraminal stenosis was not demonstrated in MR images (sagittal and transverse sections through the foramen) in four of the six patients. SRG showed that all patients had pain radiating towards the sites where pain is usually felt at the time of nerve root puncture (pain reproduction). Transiently after nerve root block, the pain dramatically disappeared, leading to clear identification of the affected nerve root. Nerve root compression by the superior articular process of the lower vertebra was detected in all patients. Representative images are shown in Figures 1–3.

## Discussion

Although the frequency of lumbar lesions in RA is not considered to be as low as previously described, their natural course has not been elucidated because there have been only a few systemic reports (1–3). This is likely due to the occasional difficulty in differentiation of RA-specific lesions from osteoporotic fractures of the vertebral body and degenerative spondylolisthesis. Moreover, complications such as cervical myelopathy and articular lesions of the lower extremities make neurological diagnosis more difficult (2).

RA-associated lumbar lesions are roughly divided into vertebral lesions, facet joint lesions, and intervertebral disc lesions. These lesions must be differentiated from spondylosis deformans and osteoporosis lesions. Vertebral lesions (defined as rheumatoid nodules of the vertebral bodies) were reported in 1952 by Baggenstoss et al. who observed them in autopsy patients (4). Facet joint lesions were described by Lawrence et al. (6). In the present study, we defined RA lumbar lesions as rheumatoid nodules of the vertebral bodies (4), vertebral collapse without previous trauma (5), disc space narrowing without osteophyte formation, end-plate erosion (2), and segmental instability associated with facet joint destruction (6,7). End-plate erosion was obvious in all of the six patients. The occurrence of RA lesions in the vertebral bodies and intervertebral disc (which has no synovial tissue) was attributed to enthesopathy by Shichikawa et al. (12). In most of the reported cases of RA lumbar lesions, neurological symptoms are caused by spinal canal stenosis, stemming from vertebral lesions leading to vertebral collapse (4,5) or from facet joint lesions leading to segmental instability (6,7). There are very few cases of foraminal stenosis. Heywood reported a case of L4 radiculopathy attributable to a L4/5 facet joint lesion (8), yet he did not describe the pathogenesis clearly.

Myelography and postmyelographic CT scans cannot be used to establish a diagnosis of foraminal stenosis, which can be detected in MRI and SRG. In four of the six cases of our series, however, MRI failed to detect foraminal stenosis. One possible reason is that the nerve root compression is not detected by recumbent MRI because the pain was relieved in the recumbent but not the sitting or standing position. In contrast, an invasive examination, SRG, detected the nerve root compression attributable to the superior articular process of the lower vertebra in all patients. Furthermore, SRG was very useful in the functional diagnosis based on pain reproduction at the time of nerve root puncture and blocking effect in RA cases where the neurological diagnosis cannot be easily established. This seemed to be a characteristic feature of RA in terms of arthritis. We assumed that the pathogenesis of foraminal stenosis in the lumbar spine of RA patients is the compression of the nerve root by the superior articular process of the lower vertebra; this is caused by vertical instability of the facet joint when facet joint lesions are associated with disorders in the vertebral body and the intervertebral disc. Impaired nerve roots due to foraminal stenosis are most frequently reported to be present at L5 in spondylosis (13), whereas L4 was the most common site for nerve root impairment in RA.

With regard to surgical treatment, Crawford et al. stated that RA patients who undergo an instrumented lumbar fusion can expect a slightly higher complication rate than patients without RA, which may be related to osteopenia and immunosuppression (3). Inaoka et al. performed posterior lumbar interbody fusion on seven RA patients and reported that collapse of graft occurred in one patient, migration of pedicle screw in two, instability of adjacent level in three, and collapse of adjacent vertebra in four (14). Fusion surgery might be performed in view of the pathogenesis of lumbar radiculopathy caused by foraminal stenosis in RA, but it was often difficult to employ fusion surgery with instrumentation because most of the patients in our series were in poor general condition or had severe osteoporosis. We performed posterior decompression without fusion in three cases; short-term outcomes were favourable though one patient was re-operated for secondary radiculopathy. Symptoms of radiculopathy were reduced by posterior decompression of the affected nerve root with partial resection of superior articular process which served as a compression factor.

## Conclusions

Lumbar foraminal stenosis is a characteristic pathology of RA, and should be kept in mind in the diagnosis of lumbar radiculopathy. SRG is useful in the diagnosis of affected nerve roots.
